# Does an L-glutamine-containing, Glucose-free, Oral Rehydration Solution Reduce Stool Output and Time to Rehydrate in Children with Acute Diarrhoea? A Double-blind Randomized Clinical Trial

**Published:** 2007-09

**Authors:** Claudia Gutiérrez, Sofía Villa, Felipe R. Mota, Juan J. Calva

**Affiliations:** 1 Teaching Department and Nephrology Department, Hospital Infantil de México Federico Gómez, Secretaráa de Salud, Mexico City, Mexico; 2 Coordinación de Investigación Epidemiológica y Servicios de Salud, Instituto Mexicano del Seguro Social, Mexico City; 3 Clinical Epidemiology Unit, Instituto Nacional de Ciencias Módicas y Nutrición Salvador Zubirán, Secretaría de Salud, Mexico City

**Keywords:** Child, Clinical trials, Dehydration, Diarrhoea, Double-blind method, Glucose, Glutamine, Oral rehydration solutions, Oral rehydration therapy, Randomized controlled trials, Rehydration, Rotavirus, Mexico

## Abstract

This study assessed whether an oral rehydration solution (ORS) in which glucose is replaced by L-glutamine (L-glutamine ORS) is more effective than the standard glucose-based rehydration solution recommended by the World Health Organization (WHO-ORS) in reducing the stool volume and time to rehydrate in acute diarrhoea. In a double-blind, randomized controlled trial in a Mexican hospital, 147 dehydrated children, aged 1–60 month(s), were assigned either to the WHO-ORS (74 children), or to the L-glutamine ORS (73 children) and followed until successful rehydration. There were no significant differences between the groups in stool output during the first four hours, time to successful rehydration, volume of ORS required for rehydration, urinary output, and vomiting. This was independent of rotavirus-associated infection. An L-glutamine-containing glucose-free ORS seems not to offer greater clinical benefit than the standard WHO-ORS in mildly-to-moderately-dehydrated children with acute non-cholera diarrhoea.

## INTRODUCTION

During the last three decades, the distribution and promotion of oral rehydration solutions (ORS) have helped save millions of lives and have been a model as a cost-effective public-health intervention, yet the standard glucose-based solution recommended by the World Health Organization (WHO-ORS) does not significantly reduce the gross stool volume nor the duration of the diarrhoeal episode. Thus, there has been a great interest in developing alternative efficacious ORS capable of reducing stool output and duration of illness, which could enhance their acceptance by users (1).

The amino acid L-glutamine is a theoretically-useful component in ORS because of its ability to act as a co-transport substrate with sodium, which leads to an increased absorption of NaCl, and its widely-known property as a fuel source for rapid turnover cells, including intestinal epithelial cells, lymphocytes, fibroblasts, and reticulocytes (2). Findings of animal and human studies have demon-strated that glutamine supplementation can ameliorate the intestinal damage and improve the gastrointestinal function and gut and systemic immune function (3). In a perfused rat ileum model exposed to cholera toxin, a dramatic reduction of toxin-induced water and sodium secretion, when a glutamine-based solution is used, has been demonstrated (3). This evidence suggests that a glutamine-based ORS might be the ideal solution to facilitate absorption of sodium and water and to improve healing from an enteric infection.

In two clinical trials among children with non-cholera diarrhoea, no advantage of the glutamine-based ORS, compared to the WHO-ORS, was found (1,4); however, the former solution contained both glucose and glutamine, and its osmolarity was 380 mOsm/L (vs 311 mOsm/L in the WHO-ORS), which may explain the observed lack of benefit from the glutamine-ORS. Glutamine is an effective co-transporter of sodium in diarrhoea but its superiority to glucose in terms of limiting stool volume has not been proved in these studies, probably due to the high osmolarity of the experimental solutions.

Because there is no published study testing the efficacy of an ORS in which glucose is replaced by L-glutamine, we were interested in testing whether this solution, with a relatively low osmolarity, could be a more effective ORS by reducing the faecal volume in children with acute diarrhoea. The main objective of this study was to assess if an L-glutamine-containing, glucose-free ORS, could reduce the stool output and the rehydration time in mildly-to-moderately dehydrated children with acute diarrhoea compared to the standard glucose-based formulation recommended by the WHO.

## MATERIALS AND METHODS

### Study design

This is a double-blind, randomized controlled clinical trial. Eligible children were randomly allocated to either of the two study arms: the L-glutamine-based solution or the glucose-based solution (WHO-ORS).

### Eligibility criteria

We included children aged 1–60 month(s) who had diarrhoea (defined as the passage of three or more loose or watery stools in the 24-hour period prior to enrollment) for not more than five days, with dehydration (with at least two of the diagnostic signs given by the WHO guidelines) and seen at the Oral Rehydration Service of the Hospital Infantil de México Federico Gómez during 1 September 2002–1 December 2003. Eligible children were screened at the hospital by a paediatrician and included in the study if the responsible caregiver signed a written informed consent to participate. A child was excluded if he or she had severe dehydration and/or hypovolemic shock, altered consciousness, dysentery, severe malnutrition (5), or high stool output (defined as ≥10 mL/kg/hour). Children were eliminated from the study when the caregiver asked for voluntary drop-out, or when the child had incomplete laboratory results.

### Baseline assessment

A thorough clinical history, which included data on family history, duration of diarrhoea, fluids ingested prior to admission of patient, number of stools and vomits in the previous 24 hours, presence of fever (≥38.0 °C), and body-weight and height. On admission, all children were weighed using a scale with 50-g sensitivity (Tanita model 1580, Tokyo, Japan). A faecal sample was obtained for identification of rotavirus, *Vibrio cholerae*, and other common enteropathogens, such as *Shigella* spp., *Salmonella* spp., and *Campylobacter jejuni*, and to determine faecal-specific gravity. A blood sample was obtained to measure urea, creatinine, glucose, sodium, potassium, and osmolality. A urine sample was obtained to measure sodium, potassium, osmolality, and specific gravity.

### Interventions

Both WHO-ORS and L-glutamine ORS were packed in identical bags, and their administration started on the day of enrollment. The WHO-ORS contained sodium chloride−3.5 g/L, dihydrated trisodic citrate−2.9 g/L, potassium chloride−1.5 g/L, and anhydride glucose−20.0 g/L and had a total osmolarity of 311 mOsm/L. The L-glutamine-based solution contained sodium chloride−3.5 g/L, dihydrated trisodic citrate−2.9 g/L, potassium chloride−1.5 g/L, and L-glutamine−20.0 g/L and had a total osmolarity of 284 mOsm/L. Both the solutions were identical in appearance and taste and were identified with a secret code (double-blinding). Additionally, both the solutions were assigned a unique serial number. The randomization list that linked serial numbers with the ORS group identity was kept at the Hospital's Pharmacy until the end of enrollment and follow-up. The study staff was blinded to the ORS assigned, while the child remained in the study. The assigned solution was given to the mother with instructions for administration to her child, along with standard messages on appropriate feeding (6-8) according to the recommendations of WHO. Both the study groups received the oral solution at an initial amount of 100 mL/kg for four hours; this was given by the patient's mother in fractionated doses every 30 minutes using cup and spoon. If vomiting occurred in excess of once per hour, oral administration was withheld for 20 minutes; if there was no abdominal complication that contraindicated oral intake, the ORS was re-introduced and increased at a dose of 0.5 mL per kg/weight every five minutes. If the child did not vomit for 10 minutes, intake was progressively increased until reaching the initial dose. If the child continued vomiting, the patient underwent rehydration using a nasogastric tube at an amount of 25–30 mL per kg of weight per hour. If vomits persisted, despite the use of the nasogastric tube, the child was rehydrated by the intravenous (IV) route.

### Follow-up and outcome measurements

All patients were clinically evaluated every hour, and data were registered. Faeces, vomits, and urine were collected in plastic bags or graduated recipients and their volume quantified every hour. The main outcome measure was the time to achieve a successful rehydration, i.e. the span between the start of oral rehydration therapy (ORT) and the moment rehydration was achieved. Rehydration was defined as when (a) the child presented none or only one of the signs of dehydration, (b) the stool output was less than 10 mL/weight kg/hour, (c) the urinary-specific gravity was less than 1,030, and (d) body-weight was stable during three consecutive hours. When this occurred, usual feeding was re-introduced, and the caregiver was trained to continue treatment at home. Two definitions of failure to rehydrate were used. One was when the child presented a stool output of ≥10 mL/weight kg/hour during four consecutive hours without improvement of the signs of hydration status and the other, when dehydration signs worsened at any moment of treatment. When the former definiton occurred, a rice starch powder solution was started at a dose of 25 mL/weight kg/hour until reduction in stool output, if no worsening of dehydration, was observed. If after another four hours, the stool volume did not decrease with the rice starch solution or if dehydration signs worsened, the child was rehydrated using the intravenous route. Osmotic diarrhoea was defined as when the faeces osmolality (Na faecal + K faecal × 2) was ≥100.

### Ethics

The trial received ethical clearance from the Ethics and Investigation Committee of the Hospital Infantil de México Federico Gómez in Mexico City. Implementation of all aspects of the project was in agreement with the International Ethical Guidelines for Research Involving Subjects, as stated in the latest version of the Helsinki Declaration. Informed and written consent was obtained from parents at the beginning of the study.

### Statistics

Statistical analyses were undertaken using the SPSS (version 10.0) statistical data management program package. All analyses were conducted on an intention-to-treat basis. The Mann-Whitney *U* test was used for determining the differences between medians, chi-square test for the comparison of proportions, and the Spearman's correlation coefficient (R_s_) in the assessment of the association between two dimensional scale values. The odds ratio (OR) and its 95% confidence interval (CI) were calculated in 2 by 2 contingency tables. The time-dependent cumulative probability of achieving a rehydration status was calculated using the Kaplan Meier analysis and the comparison between curves, according to the type of ORS, was made with the Log rank test. The proportional hazard ratio (HR) and its 95% CI of the time to a successful rehydration with the L-glutamine ORS, with the WHO-ORS as the reference, were estimated with a Cox regression model. A minimum sample size of 124 patients was calculated as required to have a power of 80% to detect as a statistically significant (two-tailed p value at 5%) difference between groups, in the stool output, of at least 30% (9). Considering the possibility of up to a 20% drop-out rate, 74 children were enrolled to be assigned to each of the two arms of the trial.

## RESULTS

In total, 148 dehydrated children with diarrhoea were enrolled into the study. Seventy-four were randomly allocated to receive the WHO-ORS and 73 the L-glutamine solution. Parents of one child in the L-glutamine ORS group decided to voluntarily drop-out from the study. Baseline characteristics at study entry were comparable between both the groups, including rotavirus and bacterial identification in faeces (p>0.05) (Table 1).

**Table 1:** Baseline characteristics of 147 enrolled children

Variable	WHO-ORS (n=74)	L-glutamine ORS (n=73)	p value
Median age (months) (minimum-maximum)	11 (1–39)	10 (2–34)	0.63∗
Male gender, no. (%)	47 (63.5)	43 (58.9)	0.56†
Nutritional status, no. (%)			
First-grade malnutrition	41 (55.4)	37 (50.6)	0.16†
Second-grade malnutrition	13 (17.6)	9 (12.3)	
Percentage of dehydration‡, median (minimum-maximum)	4.3 (1–10.8)	4.2 (2–10.2)	0.59∗
Time (hours) with diarrhoea before enrollment, median (minimum-maximum)	37 (5–120)	47 (10–120)	0.16∗
Total number of stools 24 hours before enrollment, median (minimum-maximum)	8 (1–27)	10 (2–30)	0.053∗
Total number of vomits 24 hours before enrollment, median (minimum-maximum)	6 (0–23)	6 (0–28)	0.96∗
Fever ≥38.0, no. (%)	40 (56.8)	47 (62.3)	0.20†
Antibiotic use, no. (%)	42 (56.8)	44 (60.3)	0.66†
Anti-diarrhoeic use, no. (%)	18 (24.3)	11 (15.0)	0.16†
Use of hyperosmolar fluid, no. (%)	8 (10.8)	13 (17.8)	0.22†
Reporting rotavirus in faeces, no. (%)	35 (47.3)	27 (37.0)	0.20†
*Salmonella* group D	1	1	0.99†
*Campylobacter jejuni*	0	2	0.15†
*Pseudomonas aureuginosa*	1	1	0.99†
*Shigella sonnei*	0	1	0.31†
Serum sodium level, median (mEq/L) (minimum-maximum)	140 (116–158)	138 (116–158)	0.24∗
Serum glucose level, median (mg/dL) (minimum-maximum)	92 (42–155)	90 (59–180)	0.59∗
Urinary gravity, median (minimum-maximum)	1,028 (1,025–1,030)	1,027 (1,024–1,030)	0.41∗

∗ Mann-Whitney U test;

† χ^2^ test;

‡ Weight at enrollment × 100/weight at end of rehydration ORS=Oral rehydration solution; WHO=World Health Organization

All children were successfully rehydrated with ORS and discharged alive from the Hospital. The cumulative probability of being rehydrated at 4, 6, and 10 hours in the WHO-ORS group was 26%, 64%, and 93% respectively, and in the L-glutamine-ORS group, these frequencies were 34%, 67%, and 97% respectively (Log-rank test, p=0.69). Only one patient from each group required 12 hours or more to be rehydrated. In the time to rehydrate analysis, the hazard ratio of the effect of the glutamine-based ORS, with the WHO-ORS as the reference, was calculated to be 1.06 (95% CI 0.76–1.46).

Six children (8%) in the WHO-ORS group and four children (5%) in the L-glutamine-ORS group showed failure to the original ORS, and this was changed to a rice-powder ORS (OR=0.66; 95% CI 0.15–2.79; p=0.53; with the WHO-ORS as the reference group). One patient (1.3%) in the WHO-ORS group, failed treatment due to persistent vomiting, was rehydrated with the WHO-ORS through nasogastric tube and subsequently returned to the oral route.

The median time and volume of ORS required to correct dehydration and the stool, urine, and vomit output during the rehydration period were similar between both the groups (Table 2).

**Table 2:** Main outcomes in children receiving two different oral rehydration solutions in the management of acute diarrhoea

Variable	WHO-ORS (n=74)	L-glutamine ORS (n=73)	p value
Time (hours) required for rehydration, median (minimum–maximum)	6 (3–12)	5 (3–15)	0.34∗
Volume of ORS required for rehydration‡, median (minimum–maximum)	24 (11–39)	24 (15–44)	0.72∗
Stool output during the time to rehydration‡, median (minimum–maximum)	11.35 (0–28.7)	11.8 (0.9–33.8)	0.80∗
Urinary output during the time to rehydration‡, median (minimum–maximum)	0.6 (0–4.9)	0.8 (0–6.6)	0.12∗
Patients with vomits, no. (%); median of vomits	17 (22.9)	23 (31.5)	0.24†
No./hour (minimum–maximum)	0 (0–15)	0 (0–1)	0.08∗
Median of vomits/hour‡(minimum–maximum)	0 (0–13.2)	0 (0–9.1)	0.17∗
Diagnosis of osmotic diarrhoea¶, no. (%)	37 (52.0)	24 (40.0)	0.16†

∗ Mann-Whitney U test;

† χ^2^ test;

‡ (mL/kg/h); ¶Defined as when the osmolality of (Na faecal ⩲ K faecal × 2) faeces was ≥100

ORS=Oral rehydration solution; WHO=World Health Organization

The median stool output (in mL/kg of body-weight) during the first, second, third, and fourth hour since the start of ORS was 9.75, 8.45, 10.85, and 10.65 respectively in the WHO-ORS group and 11.90, 13.70, 10.70, and 10.00 respectively in the L-glutamine ORS group. The differences between both the groups were not statistically significant. Another analysis stratifying children with and without rotavirus-associated infection showed that both the ORS treatment groups had similar volumes of stool output during these same time periods within each stratum (data not shown).

The subgroup analysis taking only children with rotavirus-associated infection (62 children) showed that the cumulative probability of being rehydrated at four, six, and eight hours in the WHO-ORS group (35 children) was 23%, 69%, and 89% respectively, and in the L-glutamine-ORS group (27 children), these frequencies were 41%, 67%, and 100 % respectively (Log-rank test, p=0.22). The HR of the effect of the glutamine-based ORS, with the WHO-ORS as the reference, was calculated to be 1.31 (95% CI 0.78–2.19). In addition, children with no rotavirus-associated infection (85 children), the cumulative probability of being rehydrated at 4, 6, and 8 hours, in the WHO-ORS group (39 children) was 28%, 59%, and 92% respectively, and in the L-glutamine-ORS group (46 children), these frequencies were 30%, 67%, and 80% respectively (Log-rank test, p=0.86). The HR of the effect of the glutamine-based ORS, with the WHO-ORS as the reference, was calculated to be 0.97 (95% CI 0.63–1.49).

No association between the volume of ingested ORS and the stool output was found in any of the two treatment groups (Rs=0.30 in the WHO-ORS group and Rs=0.53 in the L-glutamine solution group; p=0.20). The median levels of serum sodium and glucose, at the end of rehydration period, showed not to be significantly different between the groups (p=0.84 and p=0.25). No children developed hypo- or hypernatraemia or hypo- or hyperglycaemia at the end of the study. The median of serum creatinine, serum and urinary osmolality, and the proportion of patients with osmotic diarrhoea, at the end of the study, were not significantly different between both the groups (p=0.55, 0.53, 0.64, and 0.16 respectively).

## DISCUSSION

There are few clinical trials in the literature that tested L-glutamine-containing oral rehydration solutions. One trial was carried out in adults with cholera, and the other one in children with diarrhoea due to diverse aetiologic agents. The former study tested a solution with 50 mmol/L of glucose and 50 mmol/L of L-glutamine, with an osmolarity of 300 mOsm/L, and showed a marked reduction in the stool output and in the duration of the diarrhoeal episode (1). In contrast, in the second study, the solution contained 90 mmol/L of L-glutamine and 90 mmol/L of glucose, with an osmolarity of 380 mOsm/L, and showed no clinical benefit in children rehydrated with the L-glutamine-added solution, probably due to the relatively high osmolarity of this ORS (4).

Our study was designed to test the hypothesis that a water and electrolyte solution in which glucose, as co-transporter of these elements, is replaced by L-glutamine would lead to better clinical outcomes in the process of oral rehydration of children with acute non-cholera diarrhoea compared to the standard WHO-recommended glucose-containing ORS. The rationale for this hypothesis is based on two theoretical advantages of the former solution; first, the evidence from experimental models showing that supplemental glutamine can ameliorate the intestinal damage, enhance the gastrointestinal function, and reduce enteropathogens’ toxin-induced water and sodium secretion in the gut and second, that through the elimination of glucose in our experimental ORS, the osmolarity of the solution is decreased from 300 to 380 mOsm/L (in the L-glutamine plus glucose-ORS) or from 311 mOsm/L (in the glucose-containing WHO-ORS) to 284 mOsm/L (in the L-glutamine-containing, glucose-free, ORS). To our knowledge, there is no published study testing the clinical efficacy of an L-glutamine-containing, glucose-free, ORS.

The results of our study showed a similar rate of successful rehydration, time to achieve it, and stool output during the first hours of rehydration therapy, among children receiving the experimental ORS compared to those under the standard WHO-ORS.

There are several possible explanations for the lack of reduction of the stool volume and time to rehydrate in our children treated with the L-glutamine ORS. First, this amino acid might have a lesser effect on intestinal absorption of water and sodium in non-cholera diarrhoea compared to diarrhoea caused by V. cholerae, in which there is a relativelylarger secretion of sodium and chloride ions inside the intestinal lumen (10). This latter situation would provide a larger amount of sodium that is co-transported with the L-glutamine with a consequent major reduction in the stool output. These phenomena may not occur in diarrhoea secondary to other infectious agents, such as rotavirus and enteropathogenic *Escherichia coli*, as is the case with most of our study children. Second, it is widely known that rotavirus is the main cause of severe dehydrating diarrhoea, frequently associated with a high rate of stool output (11,12); thus, the inclusion of these patients in our study (42% of the total study sample) might have diluted a possible beneficial effect of the L-glutamine ORS in children with diarrhoea by other aetiologies. Yet, a stratified analysis of a subgroup of children with rotavirus-associated or rotavirus-free diarrhoea showed no differential clinical effect for the L-glutamine ORS. Third, it might be that a high intake of ORS could lead to a larger faecal volume during the rehydration period, as suggested by others (13); however, in the analysis of our own data, such association was not found.

Worth mentioning is that none of our patients developed hypoglycaemia (14), despite the fact that the experimental solution did not contain glucose. Ronan *et al*. reported transitory hyperglycaemia associated with dehydration-generated stress in patients with acute diarrhoea (15). This may explain why in our children blood glucose remained within the normal range at the end of the rehydration period.

In contrast to results of other similar studies, we included both male and female children to be able to generalize our results to the overall infant population. A potential problem with this strategy is the error in the measurement of the urine and stool volumes, as they can get mixed in female infants. However, a special care was taken in separating and quantifying urine from stool outputs in our study. Moreover, we consider that the amount of urine in these infants could have been minimal due to the low rate of diuresis that dehydrated patients present during the rehydration period.

In the present research, we did not assess the total duration of diarrhoea and stool output after discharge from the hospital, as outcome measures, because it is very difficult to reach a control of all the determinants of these events, such as the child's feeding and oral fluids administered at home. This methodological issue may limit the external validity of our results

In conclusion, the glucose-free L-glutamine ORS seems not to offer an additional clinical benefit over the standard glucose-based WHO-ORS, among dehydrated children aged less than five years with non-cholera acute diarrhoea. This lack of an additional advantage is independent of rotavirus-associated infection. Because the cost of L-glutamine-based ORS is relatively high, its indication would be limited to certain circumstances, such as an alternative rehydration therapy in children in whom the glucose-based ORS is contraindicated due to transitory carbohydrate intolerance (16,17). Further studies are needed to test a possible clinical advantage of the L-glutamine ORS in enteric illness due to particular pathogens-producing cholera-like enterotoxins.
